# Core-Shell Composite MIL-101(Cr)@TiO_2_ for Organic Dye Pollutants and Vehicle Exhaust

**DOI:** 10.3390/molecules28145530

**Published:** 2023-07-20

**Authors:** Lei Wu, Mengmeng Zhao, Xian Xin, Qiuyan Ye, Kun Zhang, Ziwei Wang

**Affiliations:** School of Materials Science & Engineering, Chang’an University, Xi’an 710064, China; z15281126833@163.com (M.Z.); 13201803828@163.com (X.X.); 15085713539@163.com (Q.Y.); z15619985393@163.com (K.Z.); qiuqiu18536062981@163.com (Z.W.)

**Keywords:** MIL-101(Cr), nano TiO_2_, photocatalytic degradation

## Abstract

MIL-101(Cr)@TiO_2_ core-shell composite material was synthesized via the hydrothermal method, where MIL-101(Cr) served as the core and TiO_2_ acted as the shell. SEM results revealed that the metal-organic framework core effectively prevented the aggregation of TiO_2_ nanoparticles and facilitated their dispersion. Characterization techniques such as XRD, XPS, and TGA were utilized to confirm the successful loading of TiO_2_ onto MIL-101(Cr) and its excellent thermal stability. MIL-101(Cr)@TiO_2_ was employed in photocatalytic degradation of dye pollutants and vehicle exhaust, and the potential degradation mechanisms were investigated in detail. The results showed that MIL-101(Cr)@TiO_2_ exhibited excellent photocatalytic degradation performance towards dye pollutants, with degradation efficiencies of 91.7% and 67.8% achieved for MB and RhB, respectively, under visible light irradiation for 90 min. Furthermore, the photocatalytic degradation of automobile exhaust revealed that the MIL-101(Cr)@TiO_2_ composite material also exhibited degradation effects on NO*_x_*, CO, and HC. The degradation efficiency for NO reached 24.2%, indicating its broader applicability.

## 1. Introduction

With the rapid expansion of industries, water and air pollution have emerged as two major challenges to human survival. Recent studies have revealed that approximately 10–20% of dyes are discharged into surface water as pollutants, which exhibit high toxicity and persist for extended periods [1,2], severely impacting the survival of aquatic organisms and humans. Concurrently, with the improvement of people’s quality of life, vehicle exhaust emissions have emerged as a significant contributor to air pollution. The harmful components present in exhaust emissions include nitrogen oxides (NO*_x_*), sulfides, carbon monoxide (CO), hydrocarbons (HC), and particulate matter (PM) [3]. These constituents are the primary causes of smog, acid rain, and global warming [4]. In view of the prevailing environmental pollution issues, diverse techniques have been developed to treat industrial wastewater and vehicle exhaust, encompassing physical, biological, and chemical approaches [2,3]. Nevertheless, these methods often suffer from drawbacks such as incomplete pollutant removal, high costs, and potential secondary pollution issues [5]. Given these circumstances, photocatalytic technology has gathered significant attention due to its simple operation, thorough purification capabilities, energy efficiency, environmental friendliness, and high effectiveness [6].

In recent times, a variety of functional nanomaterials, such as metal oxides [7,8,9,10], metal phosphates [11,12,13], metal sulfides [14,15,16,17] and carbon matrix composites [18,19,20], have been utilized as photocatalysts. Nevertheless, these material-based photocatalysts suffer from drawbacks, including low conductivity, inadequate chemical stability, and poor cycling stability [21]. Therefore, some researchers have combined metal materials with other functional nanomaterials to enhance the performance of catalysts. For example, Yu et al. [22,23] achieved excellent catalytic performance by uniformly loading Pd nanoparticles on N-doped and N/O-doped porous carbon nanospheres using an in situ thermal decomposition reduction strategy. This demonstrates that the preparation of highly efficient catalysts by combining metal nanomaterials with other functional nanomaterials is a feasible approach. Metal-Organic Frameworks (MOFs), on the other hand, are crystalline materials characterized by a high specific surface area, porous structures, and adjustable pore sizes. These novel, porous coordination polymers consist of metal ions or ion clusters combined with organic ligands [24,25,26,27]. MOFs photocatalysts possess several advantages over other semiconductor photocatalysts, including enhanced structural stability, abundant light absorption sites, and high efficiency [6,28]. Extensive research has demonstrated the excellent photocatalytic properties of MOFs. For instance, He et al. [29] reported high-performance photocatalysts based on Zr porphyrin MOFs hollow nanotubes. Ren et al. [30] demonstrated the high-photocatalytic activity of cobalt-based MOFs composites in hydrogen (H_2_) evolution. However, the photocatalytic activity of MOFs-based photocatalysts still lags behind that of noble metal-based counterparts. Furthermore, their limited conductivity and poor long-term cycling stability significantly impede their practical applications [21,31].

Hence, there has been a growing interest in the development of photocatalysts with high-catalytic performance through the integration of materials exhibiting high conductivity and MOFs. Aijaz et al. [32] utilized Pt@MIL-101 as a catalyst for gas-phase CO conversion, demonstrating its remarkable catalytic activity. Liu et al. [33] employed Pb/MIL-101(Cr) materials for catalyzing the conversion of acetone to methyl isobutyl ketone, observing a significant enhancement in catalytic efficiency upon combining Pd with MIL-101. Saikia et al. [34] prepared a composite of Fe_3_O_4_ and MIL-101 for the catalytic oxidation of benzyl alcohol, where the framework dispersion of MIL-101 materials substantially improved the catalytic performance of Fe_3_O_4_. Among these materials, TiO_2_ has been extensively studied in the field of photocatalysis; however, its limited utilization rate and weak adsorption of visible light severely restrict its application in numerous areas [3]. Consequently, the combination of TiO_2_ with MOF holds great promise for photocatalysis. Hu group [35] reported the MIL-101(Cr)@TiO_2_ photocatalyst, in which nano-TiO_2_ was successfully loaded onto the MIL-101 framework via a solvothermal method. The study focused on the adsorption and photocatalytic degradation of volatile pollutants, specifically formaldehyde and xylene. The experimental findings indicated that the composite material exhibited an augmented photocatalytic activity due to the synergistic effect. However, the study lacked an analysis of the photocatalytic degradation mechanism.

Based on the aforementioned studies, the MIL-101(Cr)@TiO_2_ composite material has been synthesized via a hydrothermal method, with MIL-101(Cr) serving as the core and TiO_2_ acting as the shell. The morphology and structure of the material were characterized using techniques such as SEM, XRD, and XPS. Additionally, the thermal stability of the composite was analyzed using TGA. Furthermore, this study provided supplementary investigations on the degradation capability of MIL-101(Cr)@TiO_2_ towards dye pollutants and automobile exhaust, along with an in-depth analysis of the degradation mechanism.

## 2. Results and Discussion

**Figure 1:**
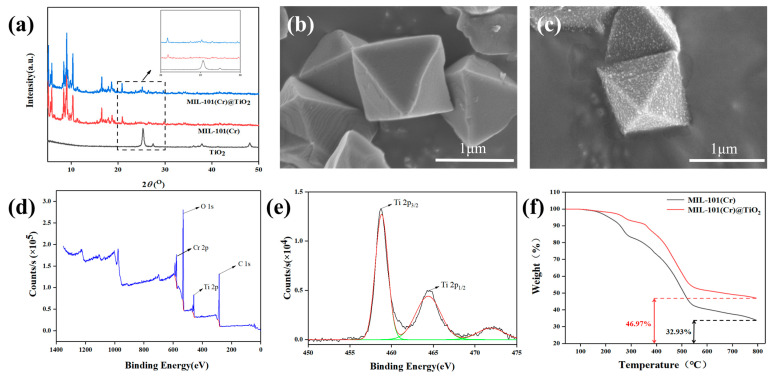
(**a**) XRD patterns of TiO_2_, MIL-101(Cr) and MIL-101(Cr)@TiO_2_; SEM images of (**b**) MIL-101(Cr) and (**c**) MIL-101(Cr)@TiO_2_; The XPS spectra: (**d**) survey scan and (**e**) Ti 2p; (**f**) TGA curves of MIL-101(Cr) and MIL-101(Cr)@TiO_2_.

The XRD patterns of the prepared MIL-101(Cr), MIL-101(Cr)@TiO_2_, and nano TiO_2_ are shown in Figure 1a. The XRD pattern of MIL-101(Cr) exhibited two sets of characteristic diffraction peaks at around 2*θ* = 5° and 10°, which were still distinctly observable in the XRD pattern of MIL-101(Cr)@TiO_2_, indicating that the incorporation of TiO_2_ did not compromise the structural integrity of MIL-101(Cr). The strongest characteristic peak of TiO_2_ is located at 2*θ* = 25.3°, corresponding to a crystal plane of (101), which was the characteristic peak and crystal plane of Rutile phase TiO_2_. In the XRD pattern of MIL-101(Cr)@TiO_2_, a faint additional diffraction peak corresponding to TiO_2_ can be observed at 2*θ* = 25°, indicating the successful formation of a composite material between nano TiO_2_ and MIL-101(Cr).

The morphology of MIL-101(Cr)@TiO_2_ composite can be distinctly observed via SEM. As depicted in Figure 1b, MIL-101(Cr) showed a regular octahedral morphology with high crystallinity and smooth surface. The uniform distribution of nano TiO_2_ on the octahedral structure of MIL-101(Cr), as shown in Figure 1c, indicated that the synthesized composite effectively addressed nanoparticle aggregation. These results demonstrated the successful preparation of the MIL-101(Cr)@TiO_2_ core-shell composite material, achieving the desired outcome.

The XPS technique was employed to analyze the chemical composition of the MIL-101(Cr)@TiO_2_. The XPS full-spectrum scan after treatment is depicted in Figure 1d, revealing the presence of chromium-, carbon-, titanium- and oxygen- peaks in the XPS survey spectra. As shown in Figure 1e, the high-resolution Ti 2p_3/2_ and Ti 2p_1/2_ peaks were detected at 458.5 and 464.6 eV, respectively, indicating the presence of tetravalent titanium in the composite material. These results demonstrate the successful loading of TiO_2_ onto MIL-101 in this experiment.

The TGA curves of MIL-101(Cr) and MIL-101(Cr)@TiO_2_ samples were presented in Figure 1f. The MIL-101(Cr) displayed an initial decrease in weight within the temperature range of 30 to 280 °C, while for MIL-101(Cr)@TiO_2_, the range was 30 to 300 °C. This weight loss was a result of the elimination of water molecules present in the guest sites. Subsequently, a substantial reduction in weight was observed until reaching a temperature of 550 °C, primarily attributed to the breakdown of the framework structure of the MOFs. Finally, the residual product of MIL-101(Cr) was considered as chromium oxide (with a residue of 32.9%), whereas the residual product of MIL-101(Cr)@TiO_2_ comprised both chromium oxide and TiO_2_ (with a residue of 47.0%). The MIL-101(Cr)@TiO_2_ composite material with a TiO_2_ loading of 14.5% was successfully prepared, as evidenced by an approximately 14.1% increase in the final solid product compared to MIL-101(Cr). Additionally, it demonstrated that an enhanced thermal stability of the composite material was achieved in comparison to bare MIL-101(Cr).

**Figure 2:**
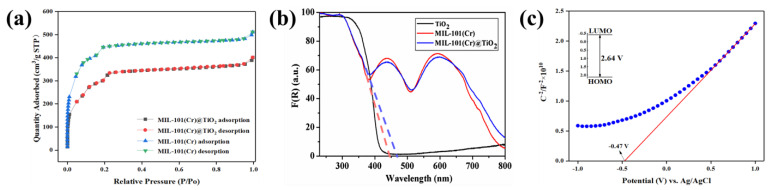
(**a**) N_2_ adsorption-desorption isotherms of MIL-101(Cr) and MIL-101(Cr)@TiO_2_; (**b**) UV-Vis DRS curves of TiO_2_, MIL-101(Cr) and MIL-101(Cr)@TiO_2_; (**c**) Typical Mott-Schottky plots (blue) and fitted line (red) of MIL-101(Cr)@TiO_2_.

The N_2_ adsorption-desorption behavior of MIL-101(Cr) and MIL-101(Cr)@TiO_2_ is shown in Figure 2a. The results indicated that the nitrogen adsorption isotherms exhibited significant adsorption behavior at low pressures, followed by a gradual flattening of the curves until reaching maximum adsorption capacity. This is a typical characteristic of Type I adsorption isotherms for microporous solids. The BET surface area and Langmuir surface area of MIL-101(Cr)@TiO_2_ were found to be 1092 and 1641 m^2^/g (micropore volume: 0.17 cm^3^/g), respectively, while those of MIL-101(Cr) were 1470 and 2111 m^2^/g (micropore volume: 0.25 cm^3^/g), respectively (Table 1). The incorporation of nano TiO_2_ onto MIL-101(Cr) resulted in a decrease in specific surface area and micropore volume, which can be attributed to the space occupied by the loaded TiO_2_, leading to a loss of partial porosity. However, MIL-101(Cr)@TiO_2_ still maintained a relatively high-pore volume, suggesting that the incorporation of nano TiO_2_ did not alter the original pore structure of the MOFs material.

**Figure 3:**
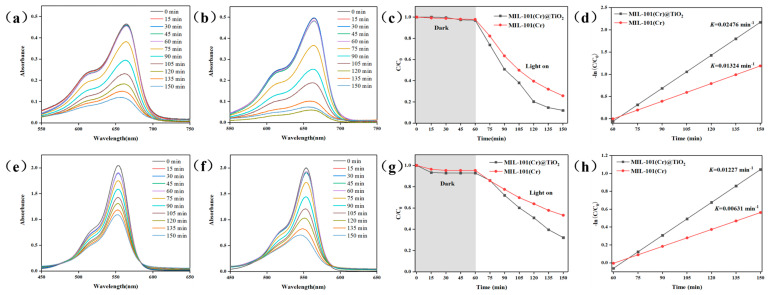
UV-Vis spectra of aqueous solutions of MB degraded by (**a**) MIL-101(Cr) and (**b**) MIL-101(Cr)@TiO_2_; UV-Vis spectra of aqueous solutions of RhB degraded by (**e**) MIL-101(Cr) and (**f**) MIL-101(Cr)@TiO_2_; Curves of *C*/*C_0_* versus time for the degradation of (**c**) MB and (**g**) RhB; pseudo first-order kinetics of (**d**) MB and (**h**) RhB photodegradation of different samples.

The optical absorption properties of TiO_2_, MIL-101(Cr), and MIL-101(Cr)@TiO_2_ samples prepared in this experiment were analyzed via DRS. The Kubelka-Munk method was used to convert the reflectance to absorbance. Figure 3b illustrates that there were no observable absorption peaks in the visible region (400–760 nm) for nano TiO_2_. On the other hand, the absorbance spectrum of MIL-101(Cr)@TiO_2_ showed two strong absorption bands at 435 and 590 nm. The optical response edge of the MIL-101(Cr)@TiO_2_ sample exhibited a shift to longer wavelengths compared to MIL-101(Cr). Additionally, the main absorption edges for MIL-101(Cr) and MIL-101(Cr)@TiO_2_ were observed at 470 nm and 440 nm, respectively, indicating band gaps (Eg) of 2.64 eV and 2.82 eV (Eg = 1240/wavelength). These findings suggest the semiconductor nature of both samples. To delve deeper into the electrochemical properties of MIL-101(Cr)@TiO_2_, a Mott-Schottky analysis was performed under dark conditions. As shown in Figure 2c, by drawing a tangent line to the longest linear section of the curve, it can be observed that the slope was positive, suggesting that MIL-101(Cr)@TiO_2_ was an n-type semiconductor [36]. Based on the x-intercept of the slanted line, the reduction potential of MIL-101(Cr)@TiO_2_ was approximately around −0.47 V vs. Ag/AgCl pH = 6.8, which is estimated to be approximately −0.27 V vs. NHE pH = 6.8. This implies that MIL-101(Cr)@TiO_2_ has the thermodynamic potential to reduce specific dyes and gaseous pollutants.

The photocatalytic degradation efficiency of MIL-101(Cr) and MIL-101(Cr)@TiO_2_ was assessed by employing MB and RhB as simulated dyes for simulating wastewater pollutants. From Figure 3c,g, it was observed that MIL-101(Cr) showed adsorption capacities of 2.2% for MB and 4.7% for RhB in the absence of light irradiation (0–60 min), whereas MIL-101(Cr)@TiO_2_ demonstrated higher adsorption capacities of 3.0% for MB and 7.2% for RhB. Subsequently, under visible light irradiation (60–150 min), the dye concentrations decreased rapidly. At 150 min, the degradation efficiency of MIL-101(Cr) reached 72.7% for MB and 46.8% for RhB, while MIL-101(Cr)@TiO_2_ achieved higher degradation efficiencies of 91.7% for MB and 67.8% for RhB. Furthermore, to systematically investigate the photocatalytic ability, the pseudo-first-order kinetic model was employed based on the following formula:

(1) $$-\ln\left(C/C_{0}\right)=Kt$$

here, *t* represents the duration of the reaction, *C_0_* denotes the concentration reached at equilibrium after 60 min, and *C* represents the concentration at time *t* within the reaction system. The relative degradation rate constant, *K*, is determined as the slope of the −ln(*C*/*C_0_*) versus time plots. Based on the information illustrated in Figure 3d,h, MIL-101(Cr)@TiO_2_ exhibited a higher *K* value compared to MIL-101(Cr), with respective values of 0.02476 (MB) and 0.01227 (RhB). The results demonstrate that the incorporation of TiO_2_ effectively enhances the photocatalytic degradation capability of MIL-101(Cr) towards dye pollutants.

**Figure 4:**
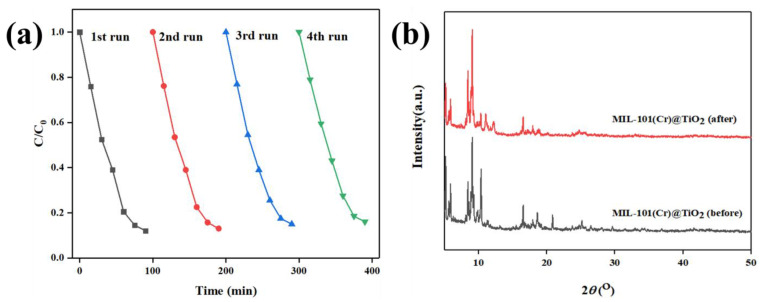
(**a**) The repeated degradation efficiency of the dye MB by MIL-101(Cr)@TiO_2_. (**b**) XRD patterns of MIL-101(Cr)@TiO_2_ photodegradation MB before and after 90 min reaction.

The durability and reusability of a catalyst are critical factors that need to be evaluated for practical applications. To assess the cyclic reusability of MIL-101(Cr)@TiO_2_, four consecutive degradation tests were conducted under identical conditions. The structural stability of MIL-101(Cr)@TiO_2_ photocatalyst during the reaction was evaluated by XRD analysis of its crystal structure. The results depicted in Figure 4a demonstrate that the degradation rate of MB remained consistently at around 90% after 90 min of visible light irradiation. However, there were slight fluctuations observed which could be attributed to the loss of a small amount of catalyst during the cleaning process prior to each repeated experiment. By comparing the XRD patterns of MIL-101(Cr)@TiO_2_ before and after the cyclic test (Figure 4b), it can be observed that the main diffraction peaks remained virtually unaltered, indicating the preservation of the crystal structure of MIL-101(Cr)@TiO_2_ after cyclic testing. In conclusion, MIL-101(Cr)@TiO_2_ exhibits excellent durability and recyclability.

**Figure 5:**
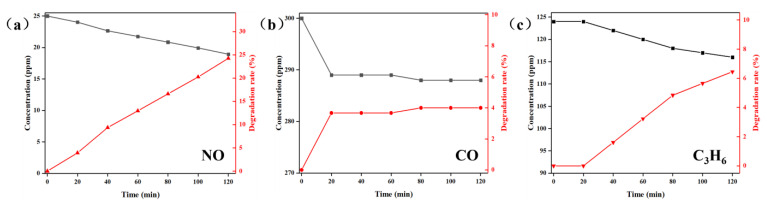
Concentration variation and degradation efficiency curves of (**a**) NO, (**b**) CO and (**c**) C_3_H_6_ under different time intervals using MIL-101(Cr)@TiO_2_ as a photocatalyst.

Figure 5 illustrates the photocatalytic degradation performance of MIL-101(Cr)@TiO_2_ towards mixed gases modeling a vehicle exhaust containing NO, CO, and C_3_H_6_. As depicted in Figure 5a, a significant decrease in NO concentration was observed within 120 min, resulting in a final degradation efficiency of 24.2% for NO. The degradation process of CO was mainly concentrated in the initial 20 min, while on the contrary, the degradation process of C_3_H_6_ mainly occurred between 20 and 120 min. However, compared to NO, there was limited degradation of CO (Figure 5b) and C_3_H_6_ (Figure 5c), with final degradation efficiency of 4.0% for CO and 6.5% for C_3_H_6_, respectively. These findings suggest that the prepared MIL-101(Cr)@TiO_2_ exhibited good photocatalytic degradation performance towards NO, but moderate degradation efficiency towards CO and C_3_H_6_.

**Figure 6:**
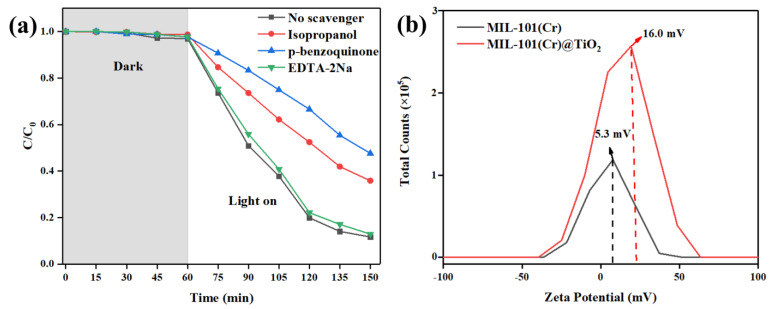
(**a**) The influence of various scavengers on the photocatalytic degradation efficiency of MB by MIL-101(Cr)@TiO_2_; (**b**) Zeta potential of MIL-101(Cr) and MIL-101(Cr)@TiO_2_.

In light of the nitrogen adsorption and desorption characteristics, MIL-101(Cr)@TiO_2_ demonstrated diminished specific surface area and porosity in comparison to the unmodified MIL-101(Cr). However, it demonstrated a higher adsorption capacity during the dark reaction stage in photocatalytic degradation tests for dyes. To investigate this phenomenon, Zeta potential testing was conducted on both MIL-101(Cr) and MIL-101(Cr)@TiO_2_. As shown in Figure 6b, the Zeta potential of MIL-101(Cr)@TiO_2_ registered at 16.0 mV, surpassing that of MIL-101(Cr) (5.3 mV). Based on Zeta potential analysis, it was observed that MIL-101(Cr)@TiO_2_ possessed stronger dye adsorption capabilities due to its higher positive potential, which was advantageous for the removal of negatively charged dye pollutants (MB and RhB) in aqueous solutions.

In order to elucidate degradation mechanism of MIL-101(Cr)@TiO_2_, active radical trapping experiments were conducted in this study, with results presented in Figure 6a. The addition of EDTA-2Na did not significantly inhibit the catalytic degradation ability of MIL-101(Cr)@TiO_2_, with an efficiency still exceeding 90%. However, the introduction of hydroxyl radicals (·OH) scavenger isopropanol and superoxide radicals (·O_2_^−^) scavenger p-benzoquinone into the system resulted in obvious decreases in the degradation efficiency of MIL-101(Cr)@TiO_2_ to 64.1% and 52.4%, respectively. These results suggest that the photocatalytic degradation process was primarily driven by ·OH and ·O_2_^−^ as the main active species, with the ·O_2_^−^ mediated redox pathway exerting a dominant influence on the reaction mechanism.

**Figure 7:**
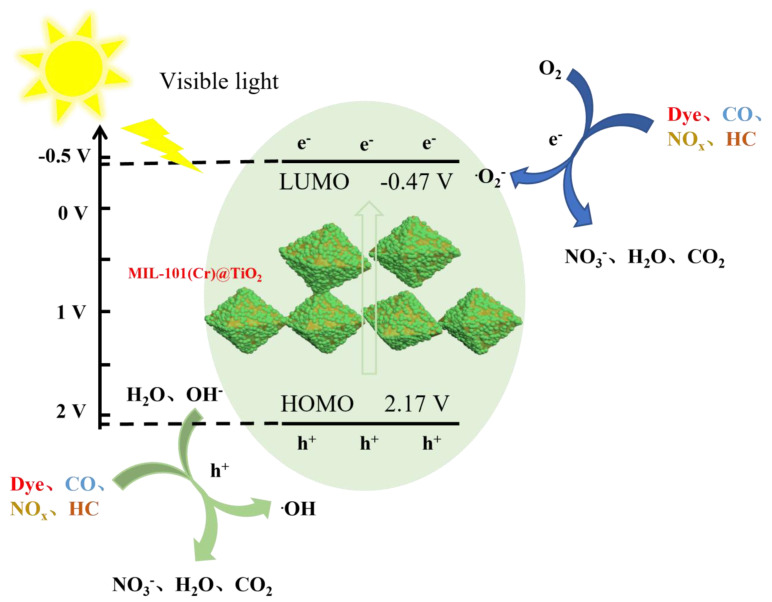
The photocatalytic degradation mechanism of MIL-101(Cr)@TiO_2_.

According to the aforementioned analysis, a potential mechanism for the photocatalytic degradation of MIL-101(Cr)@TiO_2_ can be inferred. Figure 7 illustrates the photocatalytic degradation mechanism of different pollutants using MIL-101(Cr)@TiO_2_. When exposed to visible light, both MIL-101(Cr) and TiO_2_ experienced concomitant excitation of electrons from the valence band to the conduction band. This process led to the generation of electron-hole pairs, where the valence band retained the holes (h^+^) [37]. Some of the photogenerated electrons recombine with holes either within the bulk of the composite material or at the interfaces, while another fraction migrates to the interfaces and undergoes reduction reactions by reacting with oxygen present in the system. The holes demonstrate robust oxidizing ability and can react with water and hydroxide ions within the system. The resulting ·OH and·O_2_^−^ possess highly potent oxidation capabilities, enabling the degradation and purification of organic dyes and gas pollutants such as CO, HC, and NO*_x_* into small molecules [38]. Notably, the above mechanism analysis experiment confirms that the reaction process is primarily governed by ·O_2_^−^, which assumes a pivotal role in achieving these objectives. The uniform distribution of nano TiO_2_ on the surface of MIL-101(Cr) leads to an extended path for photogenerated carrier recombination, resulting in a reduced probability of recombination and thus enhancing the catalytic degradation efficiency. The potential reaction mechanism can be expressed as follows:

MIL-101(Cr) + hv→e^−^ + h^+^

TiO_2_ + hv→e^−^ + h^+^

e^−^ + O_2_→·O_2_^−^

h^+^ + H_2_O→·OH + H^+^

h^+^ + OH^−^→·OH

·OH + organic→CO_2_ + H_2_O + mineral + acids

NO + ·OH→NO_2_ + H_2_O

NO + ·O_2_^−^→NO_3_^−^

CO + ·O_2_^−^→CO_2_

HC + ·O_2_^−^→CO_2_ + H_2_O

## 3. Materials and Methods

### 3.1. Experimental Reagents and Instruments

All the chemicals used were of reagent grade or higher quality. Chromium(III) nitrate nonahydrate (Cr(NO_3_)_3_·9H_2_O, 99%) was obtained from Tianda Chemical Reagent Factory (Tianjin, China), while isopropanol, hydrofluoric acid (HF, ≥40%), absolute ethanol, N, N-dimethylformamide (DMF, 99.5%) and tetrabutyl titanate (TBT, 99%) were purchased from Fuyu Fine Chemical Co, Ltd. (Tianjin, China). Terephthalic acid (H_2_BDC, 99%), rhodamine B (RhB, 99%) and methylene blue (MB, 98%) were purchased from Aladdin Reagent Co., Ltd. (Shanghai, China). Deionized water was self-produced.

### 3.2. Synthesis of the MIL-101(Cr)

The synthesis of MIL-101 and MIL-101(Cr)@TiO_2_ were carried out according to reference [35]. Cr(NO_3_)_3_·9H_2_O (2.4 g, 6.00 mmol), H_2_BDC (0.996 g, 6.00 mmol) and HF (0.3 mL) were added to 28.8 mL of deionized water; the mixture underwent complete dissolution with the aid of ultrasonication. Following complete mixing, the resulting solution was added to a PTFE-lined stainless-steel autoclave and underwent an 8 h reaction at 220 °C. Afterward, the reactor was allowed to cool down naturally. The resultant precipitate was then filtered and subjected to two rounds of washing, using DMF and ethanol consecutively. Lastly, the product was parched at 100 °C for 8 h in an oven.

### 3.3. Synthesis of the MIL-101(Cr)@TiO_2_

According to the literature, it has been reported that MIL-101(Cr)@TiO_2_ composite material with a TiO_2_ loading of 14.5% exhibited optimal performance [35]. Therefore, in this study, MIL-101(Cr)@TiO_2_ was prepared under the condition of a 14.5% TiO_2_ loading. MIL-101(Cr) powder (0.2 g) was introduced into a mixed solution of 50 mL TBT dispersed in 65 mL isopropanol, followed by ultrasonic treatment and magnetic stirring at room temperature for 30 min, and an aging period of 10 h. Following centrifugation, the resulting sediment underwent a thorough wash with isopropanol and was subsequently allowed to air-dry for approximately 48 h. Once the aging process was completed, the product was introduced into a high-pressure autoclave containing 70 mL of deionized water and reacted at 150 °C for 10 h. The separated samples were washed with water after cooling, followed by drying at 80 °C in an oven for 12 h.

### 3.4. Sample Activation

The samples were subjected to activation treatment to enhance the removal of residual guest molecules from the microcrystal pore cage structure of MIL-101(Cr)@TiO_2_. The prepared MIL-101(Cr)@TiO_2_ samples were activated by methanol as the displacement solvent, with fresh methanol being replaced twice a day. Afterward, the sample was heated under nitrogen protection at 150 °C for 2 h.

### 3.5. Characterization Techniques

The X-ray diffraction (XRD) pattern was acquired by a D8 ADVANCE X-ray diffractometer (Bruker Corporation, Karlsruhe, Germany). X-ray photoelectron spectrometer (XPS) was conducted using an Escalab 250Xi from Semerfeld company with a voltage of 15 kV, beam current of 15 mA, and beam spot size of 650 μm. Scanning electron microscope (SEM) images were acquired using Hitachi S4800 instrument. Thermogravimetric analysis (TGA) curves were generated using a Perkin-Elmer thermal gravimetric analyzer under conditions of 30–800 °C and a heating rate of 5 °C/min. The samples were subjected to N_2_ adsorption-desorption isotherm measurements using a Micromeritics Tristar II 3020 instrument. Prior to the tests, the samples underwent a 12 h activation process under vacuum at 200 °C. The specific surface area of the samples was measured using the Micromeritics APSP 2460 fully automated surface area analyzer. The UV-Vis diffuse reflectance spectra (UV-Vis DRS) were obtained using a UV-Vis spectrophotometer TU-1950. Spectral scanning was performed in the range of 800 nm to 230 nm with the white BaSO_4_ serving as the reference blank. Mott-Schottky measurements were carried out using a CHI760D workstation. The test employed a three-electrode system and involved four intermittent exposures to visible light. Vehicle exhaust degradation analysis was conducted using a self-designed exhaust analyzer. The zeta potential was determined via Malvern Nano ZS90 with anhydrous ethanol as the dispersion medium, and measurements were conducted under neutral conditions.

### 3.6. Photocatalytic Degradation Experiment

#### 3.6.1. Evaluation of Photodegradation Performance of Dye

The photocatalyst with a dosage of 10 mg was introduced into a 100 mL self-prepared dye solution with stirring to ensure homogeneous mixing, and incubated under dark conditions for 60 min to achieve adsorption equilibrium, followed by exposure to visible light irradiation. During the process, 4 mL aliquots of the reaction mixture were collected at 15 min intervals and centrifuged at a speed of 8000 rpm for 5 min. P-benzoquinone, Isopropanol and ethylenediaminetetraacetic acid disodium salt (EDTA-2Na) were used as scavengers (1 mmol/L) to capture ·O_2_^−^, ·OH and photogenerated holes (h^+^) in the photocatalytic process. The scavengers were added prior to the catalytic degradation experiment, followed by the initiation of dark reaction and photocatalytic degradation reaction. The resulting supernatant was tested using a UV-Vis spectrophotometer. The photocatalytic performance of the prepared material was evaluated based on the photocatalytic rate, which was calculated according to the following formula:

(2) $$\eta =\frac{C_{0}-C_{1}}{C_{0}}\times 100\%$$

where *η* represents the photocatalytic rate, *C*_0_ represents the initial concentration of organic dyes, and *C*_1_ represents the concentration of organic dyes after reaction.

#### 3.6.2. Evaluation of Photodegradation Performance of Vehicle Exhaust

**Figure 8:**
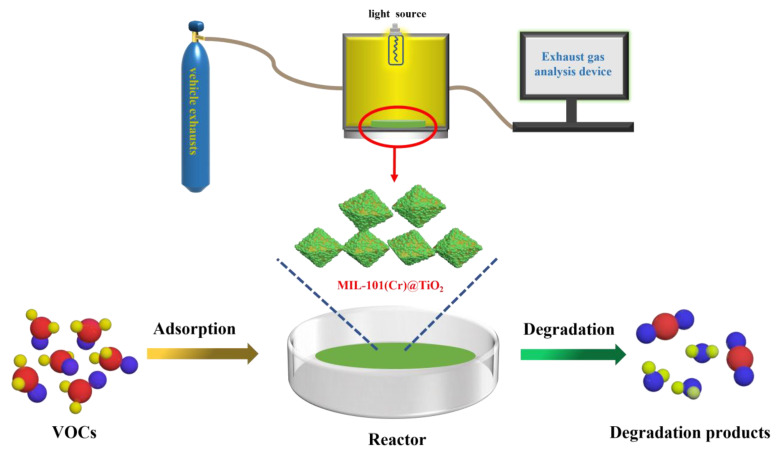
The diagram depicting the photocatalytic reaction of vehicle exhaust.

The degradation experiment of vehicle exhaust was conducted at ambient temperature using a custom-made photocatalytic vehicle exhaust reaction chamber (Figure 8). In total, 0.5 mg MIL-101(Cr)@TiO_2_ was introduced into the reactor, followed by the injection of automobile exhaust. Prior to the photocatalytic reaction, the samples were allowed to equilibrate in the reactor under dark conditions for a certain period of time until the gas concentrations reached stability. Subsequently, the reaction was carried out under visible light irradiation for a duration of 120 min. The degradation efficiency was evaluated using an exhaust gas analyzer, with gas concentrations of NO_x_, CO and C_3_H_6_ measured every 20 min to determine the changes in relation to the initial concentrations. The gas degradation rate was calculated using the following formula:

(3) $$\xi =\frac{A_{0}-A_{1}}{A_{0}}\times 100\%$$

where *ξ* represents the gas degradation rate, *A_0_* denotes the initial concentration of a specific gas in the reaction system, and *A*_1_ signifies the concentration of the gas after completion of the reaction. *A*_0_–*A*_1_ indicates the change in concentration of the degraded gas.

## 4. Conclusions

In summary, well-dispersed MIL-101(Cr)@TiO_2_ composite material was successfully synthesized. The combination of MIL-101(Cr) with nano-sized TiO_2_ extended the absorption peak of TiO_2_ into the visible light region. Through synergistic effects, the recombination of photogenerated electrons and holes was effectively suppressed, further enhancing the photocatalytic efficiency of MIL-101(Cr)@TiO_2_ composite material. The prepared MIL-101(Cr)@TiO_2_ exhibited a regular octahedral structure, high crystallinity, and good thermal stability. The performance of MIL-101(Cr)@TiO_2_ in photocatalytic degradation of dye pollutants and exhaust pollutants was evaluated, and the photocatalytic degradation mechanism of MIL-101(Cr)@TiO_2_ material was further discussed. The results showed a significant enhancement in photocatalytic degradation efficiency of MIL-101(Cr)@TiO_2_ composite material after the addition of TiO_2_. Through radical scavenging experiments, it was found that ·O_2_^−^ and ·OH were the main active species during the photocatalytic degradation process, with the oxidation-reduction pathway initiated by ·O_2_^−^ playing a dominant role in the reaction process. Additionally, cyclic degradation experiments of dyes demonstrated the good reusability of the material. This work demonstrates that MIL-101(Cr)@TiO_2_ holds great potential as an efficient multifunctional photocatalyst.

## Figures and Tables

**Table 1:** BET Surface Area, Langmuir Surface Area, and Micropore Volume of MIL-101(Cr) and MIL-101(Cr)@TiO_2_.

Sample	BET Surface Area (m^2^/g)	Langmuir Surface Area (m^2^/g)	Micropore Volume (cm^3^/g)
MIL-101(Cr)	1470	2111	0.25
MIL-101(Cr)@TiO_2_	1092	1641	0.17

## Data Availability

Data will be made available upon reasonable request.
